# Importance of adjuvant selection in tuberculosis vaccine development: Exploring basic mechanisms and clinical implications

**DOI:** 10.1016/j.jvacx.2023.100400

**Published:** 2023-10-29

**Authors:** Han Gyu Choi, Kee Woong Kwon, Sung Jae Shin

**Affiliations:** a Department of Microbiology, and Medical Science, College of Medicine, Chungnam National University, Daejeon, Republic of Korea; b Department of Microbiology, Institute for Immunology and Immunological Diseases, Graduate School of Medical Science, Brain Korea 21 Project, Yonsei University College of Medicine, Seoul 03722, South Korea

**Keywords:** *Mycobacterium tuberculosis*, Adjuvant, Next-generation vaccines, Immune correlates, Underlying disease

## Abstract

The global emergency of unexpected pathogens, exemplified by SARS-CoV-2, has emphasized the importance of vaccines in thwarting infection and curtailing the progression of severe disease. The scourge of tuberculosis (TB), emanating from the *Mycobacterium tuberculosis* (Mtb) complex, has inflicted a more profound toll in terms of mortality and morbidity than any other infectious agents prior to the SARS-CoV-2 pandemic. Despite the existence of Bacillus Calmette-Guérin (BCG), the only licensed vaccine developed a century ago, its efficacy against TB remains unsatisfactory, particularly in preventing pulmonary Mtb infections in adolescents and adults. However, collaborations between academic and industrial entities have led to a renewed impetus in the development of TB vaccines, with numerous candidates, particularly subunit vaccines with specialized adjuvants, exhibiting promising outcomes in recent clinical studies. Adjuvants are crucial in modulating optimal immunological responses, by endowing immune cells with sufficient antigen and immune signals. As exemplified by the COVID-19 vaccine landscape, the interplay between vaccine efficacy and adverse effects is of paramount importance, particularly for the elderly and individuals with underlying ailments such as diabetes and concurrent infections. In this regard, adjuvants hold the key to optimizing vaccine efficacy and safety. This review accentuates the pivotal roles of adjuvants and their underlying mechanisms in the development of TB vaccines. Furthermore, we expound on the prospects for the development of more efficacious adjuvants and their synergistic combinations for individuals in diverse states, such as aging, HIV co-infection, and diabetes, by examining the immunological alterations that arise with aging and comparing them with those observed in younger cohorts.

## 1 Introduction

The only preventive control of TB has relied on the BCG vaccine for more than 100 years since its first administration to humans in 1921 [1]. Previous studies have reported that BCG could protect children from severe TB and miliary TB [2]. It is an inexpensive, widely available vaccine that is administered to more than 90% of children in endemic countries [3]. Studies have demonstrated that BCG vaccination results in a reduction of disseminated disease and mortality in the youngest children [2]. Thus, BCG vaccine has been recommended for childhood in many countries to obtain these advantages [4].

Since its long history in clinical usage [5], the feature of BCG vaccine has been assessed to evaluate its protective roles against Mtb infection. Unfortunately, BCG vaccination has several limitations. First, accumulating evidence has demonstrated that BCG vaccination only limitedly protects against Mtb infection, with an estimated 19%, and it shows almost no protective effect in adults [5], [6]. Second, a growing number of studies have reported that BCG is protective for only 10 to 20 years from its immunization [7]. This may be the reason why the defensive efficiency of BCG in adult pulmonary TB ranges from 0 to 80% [8].

To overcome the limitations of the BCG vaccine, a variety of clinical and nonclinical investigations have been conducted for the next generation of vaccine strategies [9]. Unlike traditional vaccines that use live attenuated or inactivated pathogens, subunit vaccines use proteins, nucleic acids, and polysaccharides from pathogens. Among these subunit vaccines, protein-subunit vaccines were regarded as the novel strategy for overcoming the limitations of the BCG vaccine because of their safety in manufacture and use. Although the proteins of pathogens may act as be antigens for their host immune cells, it is necessary to use an adjuvant with protein-subunit vaccines to induce sufficient immune responses [10]. Growing evidence indicates that the selection of adjuvant is an important factor in the development of TB vaccines [11]. Next, we describe the details about the preclinical research, clinical investigation, and future perspective of adjuvants for protein-subunit vaccines against TB.

## 2 Vaccine development strategies and platforms

Historically, vaccination involved injecting healthy people with dried pus, vesicular fluid, or scabs from individuals infected with smallpox, which caused severe illness and death in some cases [12]. However, since Edward Jenner's pioneering work, numerous human vaccines have been developed and used clinically to target a wide range of viral and bacterial pathogens, and research into next-generation vaccines is ongoing [13]. It is clear that vaccines represent one of the most powerful global public health interventions in preventing infectious diseases and related mortality [14].

Traditional vaccination strategies involve administering attenuated or inactivated pathogens. For example, attenuated pathogen vaccines have been effective against measles, mumps, rubella, and poliomyelitis [15]. However, this approach cannot be used for immunocompromised individuals because their immune system might not be able to control attenuated pathogens, which can revert to a wild-type phenotype, causing severe disease. Inactivated pathogen vaccines use dead forms of the pathogen, resulting in a better safety profile than attenuated vaccines. Nevertheless, in some cases, these vaccines may not elicit the desired immune response, particularly cellular adaptive responses, making them less efficacious than attenuated vaccines in terms of vaccine efficacy but superior in terms of safety.

To overcome technical and implementation-related challenges, new vaccine approaches have been developed. Subunit vaccine development is based on the observation that it is unnecessary to administer the entire pathogen or immunogenic fragments to induce a robust immune response. Currently, TB subunit vaccines are prepared using recombinant proteins, purified from bacterial expression vectors, or formulated as naked DNA composed of recombinant plasmids encoding the Mtb antigens under the control of an eukaryotic promoter [16]. These vaccines can stimulate T cell responses to major subunit antigens and are safe, even in immunosuppressed individuals. Given that TB is prevalent in HIV-prevalent environments, the use of the adjuvanted protein subunit vaccine platform offers a significant advantage. Therefore, we provide an overview of adjuvants in TB vaccines and suggest new strategies for developing TB vaccines.

## 3 Adjuvants eliciting immune responses for TB vaccination

The administration of Mtb antigen alone is insufficient to produce a robust protective adaptive immune response, necessitating the activation of non-specific innate immune cells to induce an effective antigen-specific T cell response [11]. This is because T cells require secondary co-stimulatory signals in addition to antigen binding by the T cell receptor (TCR), which are usually provided by innate immune activation and cytokines. As a result, adjuvants are used to provide the additional signals required for T cell priming. Adjuvant selection is critical since different adjuvants stimulate the immune system in different ways, and some may not be protective, making adjuvant selection a pivotal factor in vaccine success.

Modern adjuvants have been designed to activate host immunity primarily by binding to innate immune receptors on the surface of antigen presenting cells (APCs). Adjuvants recognize and target pathogen-associated and damage-associated molecular patterns, such as toll like receptors (TLRs), C-type lectin receptors (CLRs), and NOD-like receptor (NLRs), ultimately activating downstream signaling cascades including NF-κB signaling. This, in turn, bridges innate signaling to the adaptive immune responses. The majority of current TB vaccine candidates contain adjuvants, but the exact mechanisms of many adjuvants are still unclear and may not necessarily involve a specific pattern recognition receptor (PRR).

While alum is one of the most widely used adjuvants and enhances the antibody response, it is associated with a major Th2-bias to the immune response [17]. Squalene emulsion (SE) is another adjuvant that is known to increase antibody responses by inducing strong B cell responses [18]. However, selecting the right adjuvant is crucial because different adjuvants stimulate the immune system differently, and some may not provide adequate protection. The traditional approach to TB vaccination has been to generate a robust Th1 response by using adjuvants that bind to various TLRs, such as Poly:IC, monophosphoryl-lipid A (MPLA), or CpG oligonucleotides [16], [19]. A commonly used adjuvant in many preclinical TB vaccines is a combined formulation of dimethyldioctadecyl-ammonium (DDA) and MPLA, which is effective but highly inflammatory and thus unsuitable for human use [20].

Recent research suggests that a balanced immune response consisting of Th1 and Th17 cells may be more effective in protecting against Mtb infection [21]. Mtb infection induces Th17 cells, which play a crucial role in neutrophil recruitment [22]. Furthermore, the choice of adjuvant used in T cell differentiation can also influence the immune response. For instance, CAF01, a liposomal adjuvant containing DDA and TDB, synthetic analogue of the mycobacterial cell wall, can activate C-type lectin receptors and elicit a Th17 response [23]. Similarly, cyclic dinucleotides (CDN) can activate the cGAS-STING pathway to generate long-lasting immunity, which is postulated to mimic Mtb intracellular infection [24].

In addition to recent advancements in TB vaccines, inducing an antibody response may also contribute to host-induced protection against Mtb infection. Antibodies possess functional properties that could potentially protect against Mtb, including antibody-dependent cytotoxicity and phagocytosis, among other well-characterized functions [25]. A recent study by *Irvine* et al. demonstrated that intravenous administration of BCG effectively protected 60% of rhesus macaques from Mtb infection, with a substantially up-regulated anti-lipoarabinomannan (LAM) IgM response in the plasma inversely correlated with Mtb burden in the lungs [26], [27]. Mtb-specific antibodies, including anti-LAM IgM, IgA, and IgG1 antibodies, were also negatively correlated with Mtb burden within the bronchoalveolar lavage fluid [27], indicating that IgM responses to Mtb infection, particularly LAM, play a significant role in protecting against the development of TB disease.

**Fig. 1:**
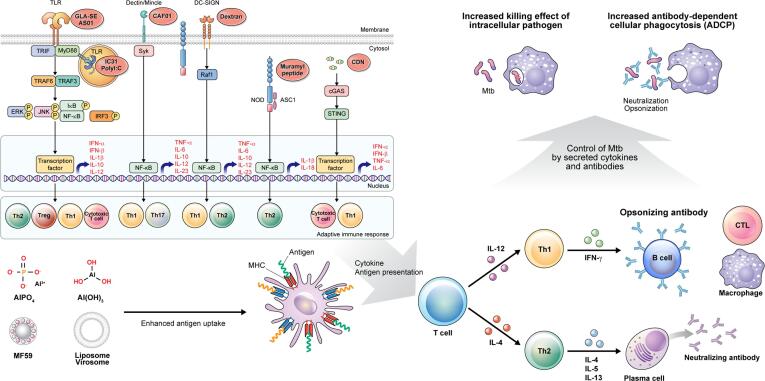
**Mechanisms of action of adjuvants for tuberculosis vaccines.** Adjuvants are immune-stimulants that intrinsically act on the immune system to improve immune responses to antigens. TLR signal through the myeloid differentiation primary response 88 (MyD88) pathway to activate NF-κB and MAP kinases or TIR domain-containing adapter-inducing IFNβ (TRIF) signaling pathway to activate IRF3, leading to secretion of pro-inflammatory, anti-inflammatory cytokines, and type I interferon. Dectins/Mincle recruit SYK1 and activate NF-κB pathway and induce cytokines driving Th1/Th17 cell differentiation. Dendritic cell-specific ICAM3-grabbing non-integrin 1 (DC-SIGN) activates NF-κB; however, the resulting gene expression is poorly understood although IL-10 expression has been shown to be induced. Nucleotide-binding oligomerization domain like receptors (NLRs) are cytosolic sensors of bacterial PAMPs to activate the NF-κB pathway and induce cytokines driving Th2 cell differentiation. The cGAS–stimulator of interferon genes (STING) pathway recognized double-stranded DNA (dsDNA) to induce the NF-κB pathway. Other adjuvants work as vaccine delivery carriers (e.g., emulsions, liposomes, or virosomes) which accurately deliver and present vaccine antigens for effective uptake by antigen-presenting cells (APC) in a controlled manner and speed to induce and/or enhance an antigen-specific immune response. In adaptive immunity, antigens combined with adjuvants are delivered to APC, which are presented by major histocompatibility complex (MHC) class I and MHC-II, thereby binding with T cell receptors on naïve CD8^+^ cells and naïve CD4^+^ T cells, respectively. Naïve CD4^+^ cells stimulate the production of Th1 or Th2 responsible for the secretion of different cytokines, and induction of cellular and humoral immunity, respectively. Induced cytokines stimulate Mtb-infected macrophages to kill intracellular pathogens, and induced antibodies can protect against Mtb infection through neutralization or opsonization. Abbreviations: TLR, Toll like receptor; NOD, nucleotide-binding oligomerization domain; TRAF, TNF receptor associated factor; ERK, extracellular-signal-regulated kinase; JNK, c-Jun N-terminal kinase; NF-κB, Nuclear Factor kappa-light-chain-enhancer of activated B cells; IFN, Interferon; IRF, Interferon regulatory factor; ASC1, activating signal cointegrator-1; Syk, Tyrosine-protein kinase; CDN, Cyclic dinucleotides.

**Fig. 2:**
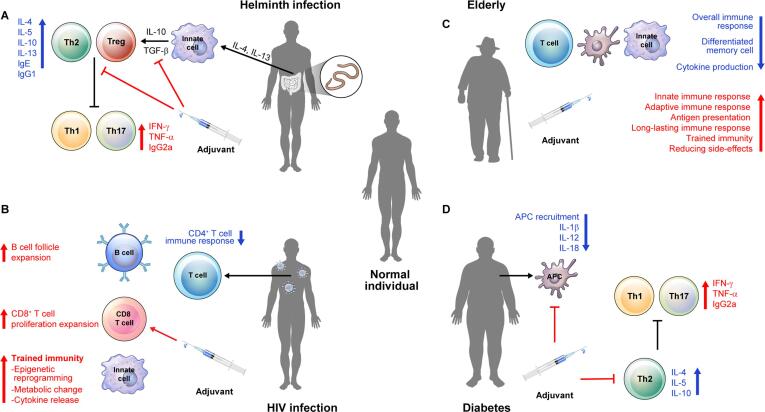
Consideration of how to use adjuvant for individuals with risk factors of tuberculosis. (A) Helminth infections induce a Th2 immune response by differentiating macrophages towards an M2 phenotype, thus polarizing the host immune system into Th2 and Treg responses (preventing Th1 or Th17 immune responses) characterized by IL-10 and TGF-β. Therefore, selecting an adjuvant that can induce M1 phenotype or Th1/Th17 response in helminth-infected individuals could be protect against Mtb, e.g., stimulate TLR-NF-κB, Dectin/Mincle or cGAS-STING signaling pathways. (B) HIV-infected individuals generally have a reduced T cell response. In particular, it is difficult to control of Mtb because the Mtb specific CD4^+^ polyfunctional or memory T cell population is reduced. Therefore, if an adjuvant that induces the expansion of B cells and CD8^+^ T cells is selected, it will be possible to protect against Mtb in HIV-infected individuals, e.g., stimulate TLR4-TRAF3, NLR or cGAS-STING signaling pathways. In particular, the use of adjuvants that can enhance trained immunity could be protect against Mtb, e.g., stimulate Dectin (β-glucan) or NLR (BCG) signaling pathway. (C) Specific changes in the T and B cell compartments occur with the onset of aging and immune senescence. Naive lymphocyte production, lymphocyte repertoire diversity, and the proliferation and functional capacity of effector lymphocytes all decrease with age. These phenomena are collectively associated with reduced TB vaccine responses and increased susceptibility to Mtb infections in the elderly. Therefore, increase vaccine acceptability by increasing immunogenicity, inducing long-lasting memory cell, and minimizing side effects should be considered when selecting an adjuvant for the elderly. Other strategies include shortening the vaccination interval or administering high doses of an adjuvant. (D) In the hyperglycemic state of diabetic patients, the function of Mtb infected alveolar macrophage is impaired, resulting in decreased expression of signals and chemokines that recruit macrophages, DCs, neutrophils, and innate lymphocytes into the lung. It also can potentially affect or reduce the frequencies of Th1 and Th17 cells in Mtb infected diabetic individuals due to an increased frequency of IL4-secreting Th2 cells. Therefore, when selecting a TB vaccine adjuvant for diabetic patients, an adjuvant capable of inducing strong activation of innate immunity and differentiation into Th1/Th17, e.g., stimulate Dectin/Mincle signaling pathway.

Mucosal immunity plays a crucial role in protection against Mtb infection, and designing a TB vaccine that takes into account the anatomical features of the lung could be of great importance [28]. One promising approach is targeting M−cells, a type of mucosal-specific cell found in nasal-associated lymphoid tissue and inducible bronchial-associated lymphoid tissue in the airways, due to their ability to rapidly transport antigens and stimulate immune responses [29]. In addition, adjuvants such as polyethyleneimine and chitosan have been utilized as penetration enhancers and immune stimulants in nasally administered vaccines, allowing for efficient access to resident APCs [30]. In the following section, we will describe various adjuvants for TB vaccine, including their molecular mechanisms, formulations, relationship with immune responses (Fig. 1), and clinical relevance based on TB risk factors (Fig. 2 and Table 1).

**Table 1:** Considerations of adjuvant selection for individuals with tuberculosis risk factors.

Risk Factors	Immunological features		Strategies for developing TB vaccine	Clinical trials for developing TB vaccine			References
	Innate immune response	Adaptive immune response		Vaccine	Phase	Outcome	
Helminthinfection	Strong systemic local type 2 immune responses Key cytokines: IL-3, IL-4, IL-5, IL-9 and IL-13 Hallmark feature: blood eosinophilia Generation of anti-inflammatory M2 macrophage by activated ILC2 Releasing of alarmin cytokines, such as IL-25, IL-33, TSLP	Induction of regulatory network Key cytokines: IL-10 and TGF-β Decreased immunogenicity Suppressed TNF-α, IFN-γ and IL-12 secretion High Treg activity High IL-4 and TGF-β secretion	(1) Modulation of immune responses Crucial to present clear Mtb antigens to increase immunogenicity Polarized Th1 Th17 response (2) Consideration Anthelmintic treatment enhance protective natural or vaccine-induced antimycobacterial immunity Identification of key factors that induces a type 2 response	BCG(Geohelminth)	Licensed	Reduced T cell proliferation and IFN-γ production Decline in Treg activity	[82], [105], [106]
				BCG(Intestinal helminth)	Licensed	Reduced the secretion of IFN-γ and IL-12 Increase in the secretion of IL-4 and TGF-β	
HIV infection	(1) Inflammasome activation NLRP3 inflammasome activated via TLR8 signaling Induction of IL-1β secretion generating pro-inflammatory immune responses Activation of dendritic cells, macrophages and NK cells Increased chemotaxis of immune cells (2) IRF3 activation Detection of PAMPs from HIV-1 by cGAS or IFI16 Activation of downstream including STING, IRF3 AND NF-κB Expression of IRF target genes, type 1 IFN and pro-inflammatory cytokines chemokines	(1) Inflammasome activation Activation of caspase 1 via ASC or NLR activation Pro-inflammatory immune responses caused by mature IL-1β Resulting pyroptosis of CD4^+^ T cell to decreased CD4^+^ T cell counts Blunted CD4^+^ T cell immune responses (2) Modulation of adaptive immune responses by NK cells Impaired antiviral T cell function via HIV-1 associated changes in DC maturation and NK cell function NK cells could kill virus-specific CD4^+^ CD8^+^ T cells and myeloid DCs	(1) Modulation of immune responses Inducing CD4^+^ T cells Expanding B cell follicles Enhancing CD8^+^ T cell proliferation Boosting trained immunity (2) Consideration Treatment status of antiretroviral therapy against HIV According to age of patients - Newborns infants children: Pre-exposure POI strategies using mRNA and DNA vaccine - Adolescent adults: Pre- and post-exposure POD strategies with subunit and non-replicating viral vectored vaccines	M72 AS01E(Subunit)	2	Higher M72-specific CD4^+^ T cells; mostly polyfunctional No CD8 detected Anti-M72 antibody peak one month after second dose (durable ∼ 3 years) Patient with antiretroviral therapy shown higher antibody titer than without HIV therapy	[89], [107], [108], [109], [110], [111], [112]
				M72 AS01E(Subunit)	1 2	M72-specific CD4^+^ T cells peak one month after second dose (durable ∼ 7 months); mostly polyfunctional No CD8 detected Anti-M72 antibody peak one month after second dose (durable ∼ 7 months)	
				H1:IC31(Subunit)	2	H1-specific CD4^+^ T cells peak one month after second dose (durable ∼ 6 months); mostly bifunctional and polyfunctional No CD8 detected Not measured H1-specific antibodies	
Elderly	(1) Inflammaging Chronic, sterile and low-grade inflammation Increased cell death rate, senescence and mitochondrial dysfunction Enhanced inflammasome and NF-κB activation Changes in hormones, ROS, nutrition, metabolism and chronic viral bacterial infections Dysbiosis of gut microbiota affecting intestinal permeability, systemic inflammation and macrophage dysfunction (2) Changes of myeloid cells Altered population of neutrophils, monocytes and DCs Reduced the function of chemotaxis, phagocytosis, signaling pathways and intracellular killing of bacteria using free radical production	(1) Changes of lymphocyte development Epigenetic and metabolic modifications; Immunosenescence Reduced primary lymphoiesis caused by changes in progenitor cells (lymphoid to myeloid) Decreased bone marrow causing decline of pro-B pre-B cells Reduced the number of naïve T B cells (2) Changes of T cells Reduced proliferative activity of T cells Gradual decline in memory and effector T cells Dysregulation of CD4 + T cell subset differentiation Basal pro-inflammatory status via increased ratio of Th17 to Treg (3) Changes of B cell maturation Increased pool of memory B cells with limited repertoire diversity Reduction of activation-induced cytidine deaminase causing decline of switched memory B cells Increased late memory B cells eliciting pro-inflammatory mediators for sustaining and propagating inflammation Increased presence of autoantibodies and low-affinity antibodies	(1) Modulation of immune responses Long-term memory T- and B-cell Induction of plasma cell (2) Boosting immunogenicity Finding new targets of antigens Increasing antigen dose Altering administration routes Use of adjuvants Developing novel adjuvants Finding the best combination between antigens and adjuvants	BCG	Licensed	Induced Th1 type immune responses Increased PPD-specific antibody	[91], [98], [113]
Diabetes	(1) Type 1 diabetes Upregulation of TLR2 4 signaling and their downstream targets, such as MyD88, TRIF and NF-κB Apoptosis of pancreatic β-cells induced by TLR3 signaling Increased levels of pro-inflammatory cytokine, reactive oxygen species and costimulatory molecules in macrophages (2) Type 2 diabetes Reduced frequency and function of NK cells with lower granulation Activation of adipose tissue macrophages promoted by increased level of IL-6 and lamin A C Increased monocyte activation Decreased the number of neutrophils and eosinophils	(1) Type 1 diabetes Generation of islet-specific CD4^+^ CD8^+^ T cells Inducing islet antigen-specific autoantibodies Hyperinflammatory phenotype of myeloid DCs could stimulate T cells via IL-12 (2) Type 2 diabetes Overactivated CD4^+^ T cells with polarization to Th1 and Th17 cells and increased secretion of TNF-α, IFN-γ and IL-17 Decreased the number of Treg cells with lower IL-10 Increased IFN-γ producing CD8^+^ T cells Stimulated proliferation of B cells via DNA methylation	(1) Modulation of immune responses Increase of chemotaxis in the infection site Boosting antigen presenting activity (2) Consideration Types of diabetes and their immunological features Monitoring blood sugar level when administering vaccine	–(Type 1 diabetes)	–	–	[102], [114], [115], [116]
	BCG(Type 2 diabetes)		Licensed	Expansion of IL-13 producing CXCR3^+^ Treg Converted proinflammatory M1 macrophages to anti-inflammatory M2 macrophage phenotype Enhanced survival and reduced inflammation			

## 4 Hijacking the TLR pathway

### 4.1 AS01E adjuvant

AS01E is an adjuvant system composed of a liposomal formulation containing a mixture of TLR4 ligand MPLA and saponin fraction QS-21 [31]. This licensed adjuvant system is already in use in the Herpes vaccine Shingrix® and contains two compounds with adjuvant properties [32]. MPL, a lipopolysaccharide derivative of *Salmonella minnesota*, is commonly used in adjuvant formulations due to its ability to activate NF-kB, bind TLR4, and induce pro-inflammatory cytokines [33], [34]. QS-21 is a natural carbohydrate-derived adjuvant purified from *Quillaja saponaria*, which binds to endosomal membrane cholesterol, disrupts lysosomal membranes, and forms pores. This leads to antigen release into the cytosol along with NLRP3 inflammasome activation, facilitating cross-presentation and activation of CD8^+^ T cells and the secretion of Th1 cytokines [19], [35], [36].

Preclinical studies have demonstrated that the Th1 polarizing effect of AS01 results from the synergistic effect of MPL and QS-21 [11]. In early stages following vaccination, subcapsular sinus macrophages in the draining lymph nodes have been observed to promote early IFN-γ production by resident NK cells and CD8^+^ T cells in a process mediated by IL-18 [37]. Analysis of blood RNA expression and antigen-specific PBMC profiles during the two-dose M72/AS01E regimen has revealed that this TB vaccine induces CD4^+^ T cells and multifunctional T cells after stimulation, although no IL-17A was detected. PBMC restimulation revealed that the vaccine induced CD4^+^ T cells and multifunctional T cells upon stimulation, although IL-17A was not detected. RNA analysis identified the upregulation of blood transcription modules associated with IFN-γ signaling, innate activation, including TLR and inflammatory signaling, as well as modules related to various chemotactic and cell adhesion processes. [38]. Recent studies have demonstrated that M72:AS01E is 54% efficacious when administered intramuscularly to HIV-negative individuals with latent TB [39].

### 4.2 IC31 adjuvant

The cationic peptide adjuvant IC31 contains the antimicrobial peptide KLKL5KLK (KLK) and ODN1a, a TLR9-binding oligodeoxynucleotide (ODN) that activates the MyD88 pathway [40]. This adjuvant may enhance ODN1a access to intracellular TLRs by stimulating endocytosis due to the cationic peptide component also functioning as an immunostimulant [41]. *In vivo* studies have shown that IC31, when administered intranasally in combination with ESAT6 antigen in a liposomal formulation, significantly reduced Mtb burden, possibly due to induction of Type I IFN activity and Th1 activity [42]. Although Type I IFN response was previously associated with disease exacerbation during development of active TB disease, in this study, it was found to be protective. IC31 is a component of two ongoing TB vaccine candidates, H4:IC31 and H56:IC31 [43], [44]. In a Phase 2b trial testing the efficacy of H4:IC31 and BCG revaccination, H4:IC31 showed only 30.5% efficacy against early or persistent Mtb infection, while BCG revaccination reduced persistent infection by 45.4% [43]. H56:IC31, on the other hand, induced specific T cell responses and generated more multifunctional memory T cells with low-dose vaccination than high-dose vaccination, which is consistent with preclinical studies that lower antigen doses may be more protective [44].

### 4.3 GLA-SE adjuvant

GLA-SE (glucopyranosyl lipid adjuvant in squalene oil-in-water emulsion) is a synthetic TLR4 agonist that promotes multifunctional immune responses via MyD88- and TRIF-dependent activation [45]. The adjuvant induces a Th1-biased immune response and relies on type I and type II IFN responses associated with IL-12 production [46]. T cell activation by GLA-SE requires IL-18 and Caspase1/11 expression, but not the NLRP3 inflammasome [47]. The ID93/GLA-SE vaccine has been tested as a prophylactic vaccine in adults vaccinated with BCG (NCT01927159) and unvaccinated (NCT01599897), and as a therapeutic vaccine in combination with antibiotics in mice and non-human primates [48], [49], [50], [51]. In humans, the ID93/GLA-SE vaccine induces the production of multi-cytokine producing T cells (TNF, IFN-γ, and IL-2) with minimal IL-17A, as well as IgG1 and IgG3 antibody production [48], [49].

## 5 Other PRR pathway mediated adjuvant

### 5.1 CAF01 adjuvant; CLR-mediated adjuvant

The adjuvant employed in the Phase 1 TB vaccine candidate H1:CAF01 is a cationic liposomal formulation composed of DDA and TDB [52]. DDA is a synthetic amphiphilic lipid that can self-assemble into vesicles, but it is unstable when used alone and tends to form aggregates. TDB is incorporated into the DDA bilayer and stabilizes the liposome [53]. TDB is highly immunostimulatory due to the activation of Mincle (Macrophage-inducible C-type lectin; primarily expressed on the surface of innate immune cells), and interestingly, when used in CAF01, it can shift the balance towards IL-17A-producing T cells [54]. These T cells contribute to protection against Mtb [55]. Thus, novel adjuvant strategies are focused on generating synthetic aryl-trehalose derivatives that provide the best Th1 and Th17 polarization [56], [57].

### 5.2 Dextran-CpG adjuvant; cGAS-STING mediated adjuvant

Adjuvants such as chitosan and CDN that activate the cGAS-STING pathway have been observed to stimulate Th1 and Th17 responses [24]. In the Phase 1 development of GamTBVac, a subunit vaccine, dextran and CpG adjuvant are used with an antigen fusion protein containing a dextran binding domain [58]. Dextran, considered “as safe” by the FDA, has been employed in medical applications as a plasma volume expander and anti-thrombotic agent, and it possesses immunogenic properties that can enhance both antibody- and cell-mediated immune responses [59]. Dextran can also interact directly with cellular receptors, such as DC-SIGN, that mediate phagocytosis. While pathogens interaction with mannose receptor and DC-SIGN may inhibit essential Th1 immune responses against intracellular pathogens [60], unlike pathogen surface molecules, dextran is an inactive ligand for mannose receptors and DC-SIGN, and it does not induce cytokine production that suppresses Th1 responses [61]. As an adjuvant, dextran may activate innate immune responses by interacting with the DC-SIGN family receptors and the mannose receptor [62]. Therefore, dextran could be an excellent adjuvant delivery system to support the deposition of mixtures of Mtb antigens fused with dextran and a mix of TLR agonists such as CpG-ODN. Currently, dextran is being used as a core material for “GamTBvac,” which is in clinical trials (NCT03878004) [58], [63].

## 6 Beyond the signaling pathway; formulations

### 6.1 Liposome based nano/microparticle

In addition to cellular activation by adjuvant itself, liposomes and emulsions are commonly used as delivery vehicles for TB vaccines. Liposomes are self-assembled nanovesicles based on lipids and are an effective delivery system capable of loading various molecules as different adjuvant formulations [20]. One of the benefits of microparticle formation is that, depending on the particle size, antigens will either be targeted to lymph nodes through drainage via the lymphatic system or will be actively transported by APCs [64]. Liposomes are effective as adjuvants, as loaded antigens are slowly released after injection, and their vesicular structure protects enclosed antigens from degradation while forming a depot [65]. Additionally, negatively charged cationic liposomes can aggregate and bind positively charged antigens, further enhancing the depot effect [66]. Therefore, both nanoparticles and microparticles are popular strategies to specifically target cell populations based on particle size, surface chemistry, and administration site [67].

Poly DL-lactic-co-glycolic acid (PLGA) is a common polymer used, which like dextran, has been used for a long time prior to adjuvant applications and is already approved for parenteral use for sustained drug delivery by the FDA [67]. Depending on surfactant and polymer concentrations and homogenization speed, PLGA can be made into nanoparticles or microparticles. There has been much interest in using PLGA microparticles for the delivery of anti-TB vaccines and treatments [68]. When administered intramuscularly as a single dose with KLK encapsulated in ∼ 7 μm PLGA microspheres, mycobacterial Hsp65 protein was highly protective [69].

### 6.2 Natural polysaccharides with inulin

Polysaccharides have been gaining attention as adjuvants due to their biocompatibility, biodegradability, and innate immune-modulatory properties [70]. Natural polysaccharides are capable of activating various immune cells, such as macrophages, T cells, and B cells, leading to downstream expression of chemokines and cytokines [70].

Advax™ is a new adjuvant made from inulin particles derived from the roots of the *Compositae* plant and has been shown to enhance vaccine immunity against several diseases when formulated as delta inulin particles [71]. Inulin has been used in medicine to measure glomerular filtration, and its insoluble fraction was found to activate complement [72], leading to the identification of an alternative complement pathway [73]. Inulin in its delta isoform forms water-insoluble cationic particles of approximately 2 µm in diameter at 50 °C, and when administered subcutaneously or intramuscularly with CysVac2 antigen, it induces a robust multifunctional CD4^+^ T cell response and protection against Mtb challenge [74]. Among its immunological effects, Advax™ induces a potent chemotactic effect, recruiting leukocytes to the site of vaccination and stimulating a wide range of immune responses to co-administered antigens, including humoral and Th1, Th2, and Th17 responses [74], [75], [76].

## 7 Vaccine adjuvants for individuals with TB risk factors

Various TB risk factors (Helminth, HIV, Age, Diabetes) suppress the host's immune response and are known to affect immunogenicity and vaccine efficacy. However, there is no systematic summary of the immunological mechanisms involved by which these risk factors affect vaccine efficacy. This section aims to the underlying immunological mechanisms of risk factors (Fig. 2) and highlight considerations in the selection of adjuvants for individuals at risk of TB (Table 1).

### 7.1 Helminth infection

Helminth infections are known to trigger a Th2-related immune response, characterized by the presence of interleukins IL-4, IL-5, IL-9, IL-10, and IL-13, and IgE, IgG1, and IgG4 production [77], [78]. While earlier studies had identified CD4^+^ Th2 cells as the main source of these cytokines, more recent research has shown that eosinophils, basophils, and innate lymphoid cells (ILCs) are also responsible for their production in response to helminth infection [78]. However, immunity to Mtb infection requires a predominant Th1 response, with contributions from Th17 cells and other helper cells. Additionally, TB is typically characterized by a strongly pro-inflammatory environment. Individuals with helminth-TB co-infection often exhibit more advanced TB disease compared to TB patients without helminth infection [79]. Furthermore, helminth infections interfere with vaccine-induced responses to TB, which is the primary reason for the reduced effectiveness of BCG in helminth endemic regions of the world [80], [81]. It has been reported that cellular immune responses to Mtb antigens are reduced in individuals with concurrent helminth infections, at least partially explaining the reduced efficacy of BCG in these regions [80]. Recent studies have demonstrated that concomitant helminth infection significantly impairs the immunogenicity of the BCG vaccine, an impairment associated with increased TGF-β production [82]. Therefore, to design a successful strategy for TB vaccine during parasitic infection, it is crucial to present clear Mtb antigens and select an adjuvant that can polarize the immune response towards Th1/Th17 (Fig. 2A).

### 7.2 HIV infection

TB vaccines face a significant obstacle in HIV-positive individuals. The immunosuppression caused by HIV infection reduces the immunogenicity and efficacy of TB vaccines, which primarily enhance Th1-type T cell immunity [83]. HIV co-infection also specifically depletes Mtb-specific IFN-γ^+^IL-2-TNF-α^+^ CD4^+^ T cells [84], [85], [86], impeding the proliferative function of T cell effectors, long-term memory, and tissue homing capacity necessary for functional TB protection and vaccine development [84], [87]. Moreover, HIV infection is a major underlying factor for active TB from LTBI, making individuals with both LTBI and HIV co-infection more susceptible to TB reactivation [84]. Recent studies have revealed additional mechanisms, such as chronic immune activation [88], expanded B cell follicles, and CD8^+^ T cell proliferation [89], that play a critical role in SIV-induced LTBI reactivation in macaques, independent of CD4^+^ T cell depletion. Some studies have hypothesized that an AMTB-SIV vaccine can induce trained immunity, a memory type of the innate immune system, which can enhance CD4^+^ T cell activation and increase SIV susceptibility in infant macaques [90]. Therefore, developing an adjuvant that can increase the immunological factors mentioned above, as well as induce trained immunity, along with using a well-presented TB antigen, may improve treatment and vaccine efficacy for TB in HIV-infected patients (Fig. 2B).

### 7.3 Older adults

As life expectancy continues to increase in developed nations, the elderly population, who are defined as individuals over 60 years old, are becoming increasingly vulnerable to infections, including SARS-CoV-2. The aging process affects various functions of the body, including the immune system, which undergoes changes collectively referred to as immune aging. As a result, older individuals are more susceptible to infections and are often less protected by vaccines. The vaccine currently used for the elderly is an adjuvant-antigen combination that has been proven effective [91]. Approved adjuvants for use in the elderly include MF59 and AS03 (seasonal influenza vaccine adjuvants), CpG (hepatitis B vaccine adjuvant), and AS01 (Herpes/Zoster vaccine adjuvant) [92], [93], [94], [95], [96].

However, there have been limited studies on the use of potent antigen delivery systems and immunostimulatory adjuvants to combat age-related deficiencies in the immune response, particularly in relation to TB vaccines. To develop an effective TB vaccine for the elderly, various strategies can be employed to increase vaccine efficacy, such as boosting immunogenicity, inducing long-lasting memory T, B, and plasma cells, and minimizing adverse effects. Additionally, older individuals exhibit a reduced response and shorter duration of protective immunity after booster immunization, thus shortened vaccination intervals could be a viable strategy to counteract the accelerated loss of protective immunity levels after vaccination.

Another consideration for increasing vaccine efficacy is sex, obesity, and the composition of the T cell repertoire [97]. Furthermore, many clinically used adjuvants show signs of inflammation in the first few hours after vaccination [98]. It is important to determine whether adjuvants should avoid increasing this inflammatory state or whether more potent adjuvants with strong inflammatory activity are required to induce an effective immune response in the elderly despite the baseline low-grade inflammatory state. Adjuvants should ideally be designed to achieve an optimal balance between immune stimulation and the inflammatory state of the elderly immune system. Therefore, standardized and systematic approaches to selecting the optimal adjuvant type and dose in the early preclinical stages of vaccine development, including the potential use of age-specific human *in vitro* and animal models, should be considered to accelerate the translation and discovery of safe and effective adjuvanted vaccines tailored for immunologically distinct populations, such as older adults (Fig. 2C).

### 7.4 Diabetes

Diabetes is a significant risk factor for developing active TB. The immune system's defective cell activation causes impaired bacterial recognition, phagocytic activity, and chemokine and cytokine production, leading to increased susceptibility to TB in diabetic patients. In hyperglycemic hosts, the initiation of adaptive immunity is delayed due to impaired APC recruitment and function, leading to reduced frequencies of Th1, and Th17 cells, macrophages, and inflammatory responses in TB [99]. An impaired immune response and killing of intracellular bacteria potentially increases bacterial load, chronic inflammation, and central necrosis, promoting bacterial dissemination. Studies in hyperglycemic mice and normoglycemic control mice exposed to aerosol challenge have revealed that Mtb-infected alveolar macrophages' function is impaired in hyperglycemic mice, resulting in reduced expression of chemokines and signals recruiting macrophages, DCs, neutrophils, and innate lymphocytes. This creates a barrier to leukocyte migration into airspace, even when appropriate recruitment signals are generated by infected alveolar macrophages [100], [101]. Reduced secretion of IL-1β, IL-12, and IL-18 and a decreased IFN-γ response to stimulation in type 2 diabetic patients may result in increased susceptibility to TB [102], [103]. In conclusion, diabetic patients have abnormal function in both innate and acquired immune responses, such as Th1 and Th17 responses, increasing the risk of complex TB development, complications, treatment failure, and death. Therefore, comprehending these immunological complexities is essential to guide TB treatment and vaccines successfully (Fig. 2D). A deeper understanding is clearly needed to select an adjuvant for TB vaccine for individual with these risk factors (Table 1).

## 8 Conclusion

Adjuvants in TB subunit vaccine formulations play a crucial role in the success or failure of the vaccine by modulating the immune response and optimizing antigen presentation. However, TB vaccine candidates have failed in the past because they selectively induce only Th1 responses. Some researchers have explored the possibility of inducing Th17 and antibody humoral responses as a new perspective for TB vaccines [104]. In the future, synergistic effects between two or more adjuvants may stimulate different innate immune pathways. Adjuvant systems utilizing two or more adjuvants in a vaccine candidate have already been introduced in TB vaccine pipelines. This review provides insight into adjuvants used in preclinical and clinical studies to find new effective TB vaccines and their importance in adjuvant selection for individuals susceptible to TB. Although the general trend is to develop adjuvants that initiate a robust and sustained Th1 response with the production of multifunctional cells, the review also presents examples of adjuvant formulations focused on Th17 and other responses such as antibody humoral responses. Additionally, individuals susceptible to TB have an abnormal immune response, and the response to vaccination varies greatly among individuals. Therefore, more specific and potent adjuvants may be needed to fine-tune the immune response and selectively stimulate pathways leading to long-lasting immune protection for individuals at risk of TB. Understanding the need for new individualized approaches may lead to the development of more effective TB vaccines. In conclusion, the development of effective TB vaccines is crucial to combat this global health challenge. Adjuvants play a crucial role in TB subunit vaccine formulations, and their selection is important in inducing a robust and sustained immune response. While the focus has been on Th1 responses, inducing Th17 and antibody humoral responses may be a promising avenue for TB vaccines. Future research on the synergistic effects of adjuvants and the development of individualized approaches may lead to more effective TB vaccines and ultimately reduce the burden of this devastating disease.

## Declaration of Competing Interest

The authors declare that they have no known competing financial interests or personal relationships that could have appeared to influence the work reported in this paper.

## Data Availability

No data was used for the research described in the article.
